# Mechanism of polygonum capitatum intervention in pulmonary nodule based on network pharmacology and molecular docking technology

**DOI:** 10.1097/MD.0000000000038419

**Published:** 2024-06-21

**Authors:** Yilian Tang, Xiang Pu, Zhiliang Fan, Xiangyan Kong, Chen Zhang, Lailai Li

**Affiliations:** a Guizhou University of Traditional Chinese Medicine, Guiyang City, China; b Zunyi Medical and Pharmaceutical College, Zunyi City, China.

**Keywords:** molecular docking, network pharmacology, polygonum capitatum, pulmonary nodule

## Abstract

The present study utilizes network pharmacology and molecular docking methodologies to investigate the mechanism of action behind the intervention of *Polygonum capitatum* Buch.-Ham.ex D. Don (THL) in treating pulmonary nodules (PN). This research aims to provide a theoretical foundation for broadening the clinical application of THL. Active components of THL were identified and screened through an extensive literature review and the PharmMapper database, followed by an analysis of their target interactions. Relevant targets associated with PN were selected using databases such as OMIM and GeneCards, with an intersection of the two sets being determined. STRING11.5 facilitated the acquisition of protein-protein interaction data, which was then imported into Cytoscape 3.7.2 to establish a protein interaction network topology. This enabled the identification of pivotal targets affected by THL intervention in PN. The study further employed the Metascape database to conduct GO and KEGG bioinformatics enrichment analyses, which illuminated core pathways involved in THL’s therapeutic effects on PN. A comprehensive component-target-pathway diagram was constructed utilizing Cytoscape 3.7.2 software, with molecular docking validations carried out via Maestro software. A total of 49 active THL ingredients were discerned, implicating 67 PN-relevant targets. Subsequent software analysis pinpointed 10 key targets, including ALB, EGFR, and SRC. Molecular docking studies indicated strong binding affinities for most protein-compound pairs, with 44 out of 60 docking results exhibiting binding energies below −5 kcal/mol. Enrichment analysis highlights that key targets are mainly involved in pathways such as cancer, lipid metabolism and atherosclerosis, estrogen signaling, IL-17 signaling, complement and coagulation cascades, and chemical carcinogenesis through receptor activation. Through comprehensive network pharmacological approaches, this research delineates the synergy of THL’s multiple components, targets, and pathways in mitigating PN. It posits that primary active ingredients of THL – quercetin, salidroside, and oleanolic acid – may exert effects on targets like ALB, EGFR, SRC, potentially modulating pathways associated with cancer, lipid and atherosclerosis, and IL-17 signaling in the context of PN intervention.

## 1. Introduction

Pulmonary nodule (PN) refers to a focal, round-like, dense solid or sub-solid lung shadow with a diameter ≤ 3 cm in lung imaging. Its pathological properties include chronic inflammation, adenomatous hyperplasia and tumor. The detection rate of PN globally has seen a dramatic rise due to the advancement and widespread utilization of imaging diagnosis technology. Modern medicine advocates for regular follow-up or surgical treatment, but lacks effective prevention and treatment drugs. In the long-term CT follow-up process, the risk of malignant transformation imposes great psychological and economic burden on patients,^[1]^ while surgical treatment has a considerable impact on the life quality of patients. Therefore, it is essential to investigate new and effective treatment methods.

*Polygonum capitatum* Buch.-Ham. ex D. Don (THL) is a defining ethnic medicine of the Miao people, and is the main of raw material for such as Sijicao Granules and Relinqing Granules. In 2020, the Guizhou Provincial Administration of Traditional Chinese Medicine put forward Relinqing Granules as a clinical remedy for treating infectious pneumonia in COVID-19 of Guizhou Province. As revealed by studies, THL extract has anti-inflammatory activity. It mainly inhibits the release of inflammatory factors by affecting the NF-κB pathway, regulate p38MAPK, Bcl-2 and Bax gene levelsand and influencing the phosphorylation of IκBα, NF-κB p65, p38MAPK, and ERK1/2 as well as NF-κB p65 and p-p38MAPK’s nuclear translocation develop the anti-inflammatory role. Unfortunately, there is a dearth of study on the pharmacological mechanism of THL intervention in Pulmonary nodule.

Network pharmacology combines multiple disciplines such as pharmacology and bioinformatics with system network analysis, Unveiling the links between traditional Chinese medicine and ailments, as well as between active components and genes, this can forecast the potential pathways of Traditional Chinese Medicine in treating illnesses, thereby furnishing a significant theoretical foundation for uncovering the pharmacological functions and mechanisms of traditional Chinese medicine. In this study, network pharmacology and molecular docking methods were used to find the multi-component, multi-target and multi-pathway relationship of THL, construct the drug component-target network diagram, and explore the material basis and possible molecular mechanism of THL intervention in pulmonary nodules from multiple perspectives, for future Research on THL treatment of pulmonary nodules as a reference.

## 2. Materials and methods

### 2.1. Active ingredient and target screening

The chemical composition of THL was summarized by referring to the related literature, and the structure of each compound was drawn by chemdraw software. Input the structure of each compound drawn into SwissADME database, and specify the criteria for selecting potential compounds as follows: 1. GIabsortion is “High,” indicating that the component has good oral bioavailability and is easy to absorb; 2. Lipinski, Ghose, Veber, Egan and Muegge have no less than 2 “Yes” in the test results.

According to the literature review results, if the pharmacological effects of compounds were highly correlated with PN, they still were included and the final potential compounds were obtained. Based on pharmacophore matching method, the core action targets of different potential compounds were obtained by using PharmmMapper database. Moreover, the target proteins with NF (NormalizedFitScore) ≥ 0.9 were selected. This study obtained the core action targets of active compounds by combining related literature reports.

### 2.2. Screening of disease targets

To guarantee the accuracy and overall of the target data, the term “Pulmonary nodule” was employed as the core keyword of PN, and In the GeneCards database (https://www.genecards.org/) And OMIM database (https://www.omim.org) Conduct a search to identify the core targets of PN disease. Then, the collected target information was deduplicated and combined. Using the UniProt protein database to standardize disease targets and potential targets for drug chemical components as Gene Symbols. Then, the two were mapped to get the potential target of THL intervention in PN.

### 2.3. THL component-PN target

The common target of drugs and diseases is input into the STRING database (http://string-db.org),^[2]^ and the biological kind is set as “Homo sapiens,” with a confidence of 0.4, constructs protein interaction (PPI) networks. Import the result into CytoScape3.7.2 software. This software can be employed to investigate protein interactions and obtain the latent functions of the pertinent proteins.

### 2.4. The enrichment of GO function and KEGG pathway

Import intersection targets into the Metascape database (http://www.metascape.org),^[3]^ select the species as “Homo Sapiens,” screen the obtained results with a critical value of *P* ≤ .01, and perform gene ontology (GO) function and KEGG pathway enrichment analysis respectively. GO function includes three parts: biological process, cellular component, and molecular function. Select the top 20 GO enrichment analysis results and KEGG pathway for visualization based on the *P* value sorting of each part.

### 2.5. Component-target-pathway network

Integrating the bioinformatics enrichment analysis results from “1.4” and the drug disease intersection targets from “1.2,” a drug component-target-pathway network diagram was created using Cytoscape3.7.2.2, the topological analysis results of potential chemical components and targets of THL, including Betweenness, Degree, etc., were obtained. Based on the results of network topology analysis, the core target and the main active ingredients of PN intervention were obtained.

### 2.6. Molecular docking evaluation

Selective analysis to obtain the medium value of THL composition-PF target network diagram ranked in the top 6. The protein pdb format was downloaded from RCSB database (https://www.rcsb.org/), and the core compounds were converted to pdb format with the help of Open Babel software. With pymol and Autodock Tools, the proteins and compounds were treated with dehydrating, deligand and hydrogenation, and stored in pdbqt format. The potential compounds are treated by reducing energy and assigning ligand atomic types and stored in pdbqt format. Autodock software was used for molecular docking processing, binding energy Affinity was used as a reference to judge the binding activity of the two, and the docking score is displayed in a heatmap. The optimal docking conformation of the binding between compounds and target proteins was visualized using PyMoL 2.1 software.

## 3. Consequence

### 3.1. THL Predictive results of main active ingredient targets

**Figure 1.**
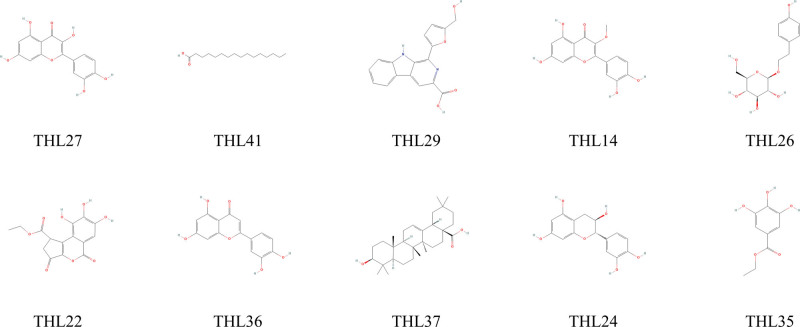
Structure of the 10 most active components in polygonum capitatum.

A total of 100 chemical structures related to THL had been obtained by synthesizing related literatures. The main components were flavonoids, lignans and volatile oils, as well as terpenoids and tannins. 49 active ingredients were screened by SwissADME, among which the structures of 10 active ingredients with the strongest activity are shown in Table 1 and Figure 1. PharmMapper was used to predict and select targets with NF ≥ 0.9, and 114 predicted targets were obtained after removal of all duplicate genes.

**Table 1:** The 10 most active components in polygonum capitatum.

Code	Compound	CAS	OB	DL
THL27	Quercetin	117-39-5	46.43	0.28
THL41	Palmitate	57-10-3	19.30	0.10
THL29	Flazin	100041-05-2	94.28	0.39
THL14	3-O-Methylquercetin	1486-70-0	10.10	0.30
THL26	Salidroside	10338-51-9	15.96	0.20
THL22	Ethyl brevifolincarboxylate	107646-82-2	30.86	0.33
THL36	Luteolin	491-70-3	36.16	0.25
THL37	Oleanic acid	508-02-1	29.02	0.76
THL24	catechin	18829-70-4	54.83	0.24
THL35	Ethyl gallate	831-61-8	25.61	0.06

THL = *Polygonum capitatum* Buch.-Ham. ex D. Don.

### 3.2. Acquisition of THL intervention in PN-related target

**Figure 2.**
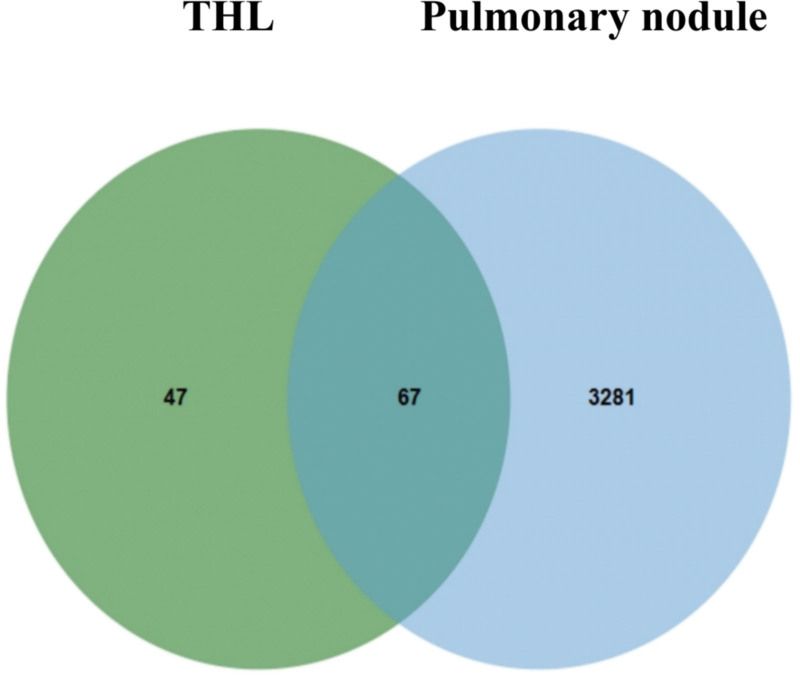
Venn diagram of the target of THL and the target of pulmonary nodule. THL = *Polygonum capitatum* Buch.-Ham. ex D. Don.

In this study, 3344 pn disease-related targets were obtained from the Genecards database, supplemented by the OMIM database and de-merged. Results 3348 targets related to pn disease were collected. 114 Intersecting THL active component targets with 3348 PN-related targets, and 67 intersecting targets were attained. The results are plotted as VENN diagram (Fig. 2).

### 3.3. Construction of PPI network

**Figure 3.**
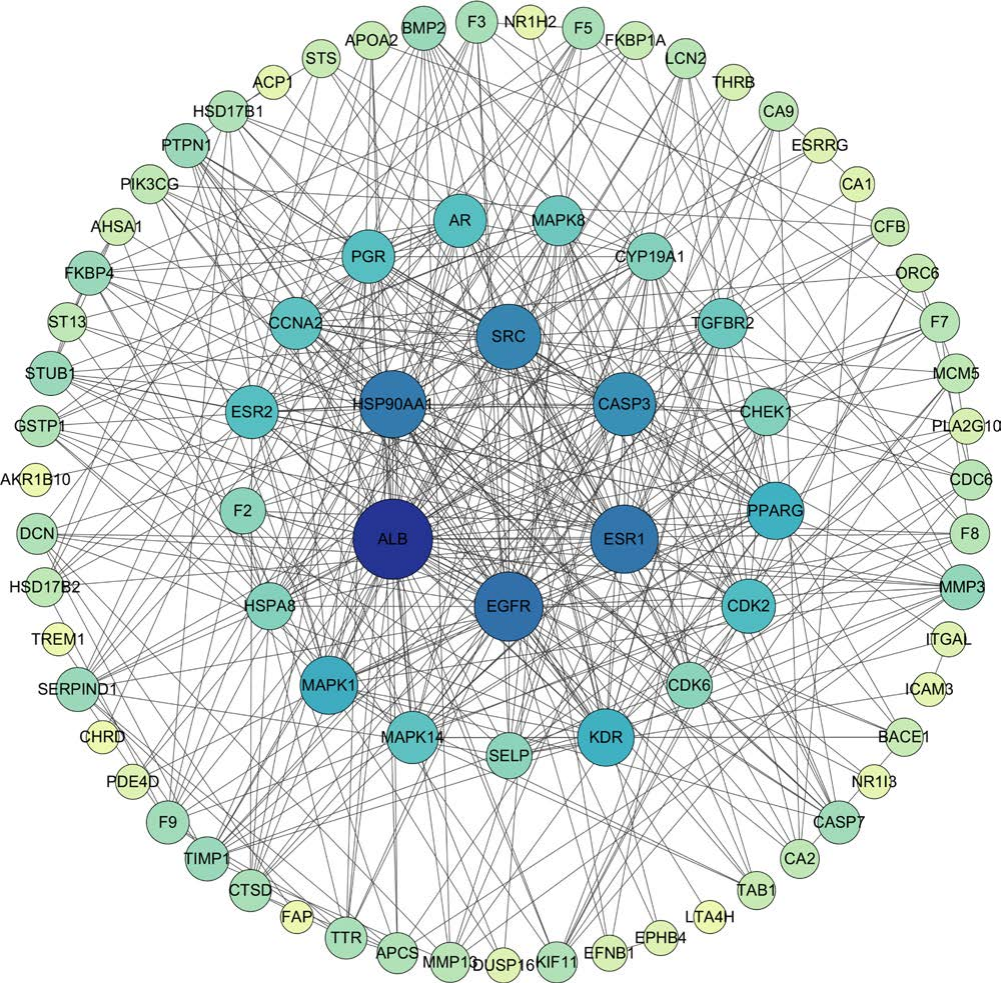
The PPI network of THL and Pulmonary nodule targets. PPI = protein-protein interaction, THL = *Polygonum capitatum* Buch.-Ham. ex D. Don.

In this study, 67 intersection targets were uploaded to String 11.5 database for PPI analysis. Utilizing cytoscape 3.7.2 software (Fig. 3) to construct the PPI network and to further analyze network information. It was speculated that albumin (ALB), Epidermal growth factor receptor (EGFR), ESR1, and heat shock protein HSP 90-alpha (HSP90AA1) were the core targets.

### 3.4. GO function enrichment and KEGG pathway enrichment

**Figure 4.**
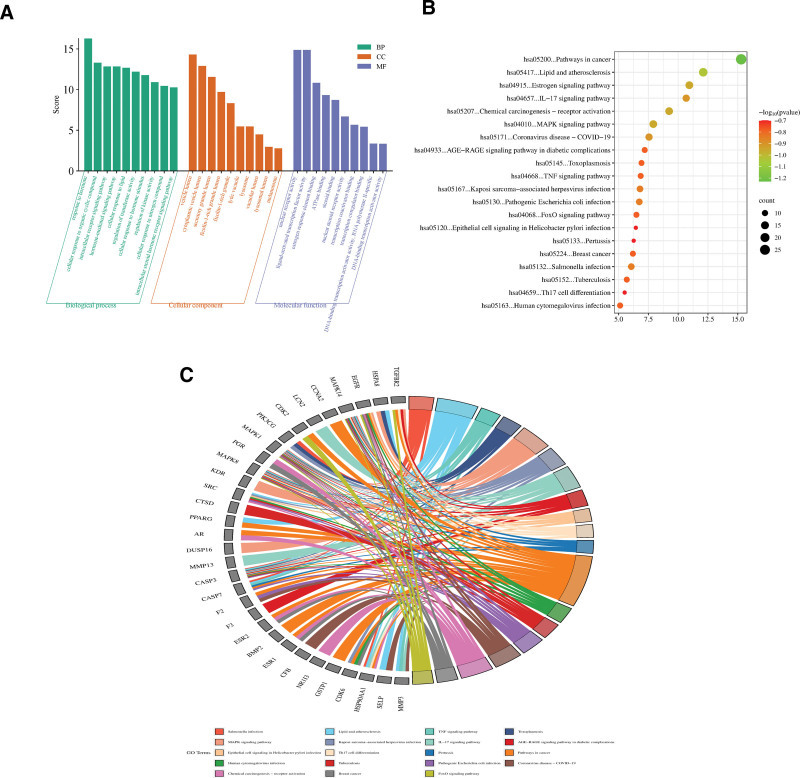
Enrichment analysis of potential targets for the main components of Polygonum capitatum (A: GO analysis; B and C: KEGG analysis). GO = gene ontology, KEGG = Kyoto encyclopedia of genes and genomes, THL = *Polygonum capitatum* Buch.-Ham. ex D. Don.

Bioinformatics enrichment analysis of potential core targets using the Metascape database. In Figure 4A THL intervene PN-related fundamental biological processes is revealed: hormone-related response, cellular response to organic cyclic compound, intracellular receptor signaling, hormone-mediated signaling, and cellular response to lipid. Figure 4B and C show the top 20 signaling pathways with THL intervention PN in the KEGG pathway enrichment analysis results.

### 3.5. Construction of THL component-target-pathway network

**Figure 5.**
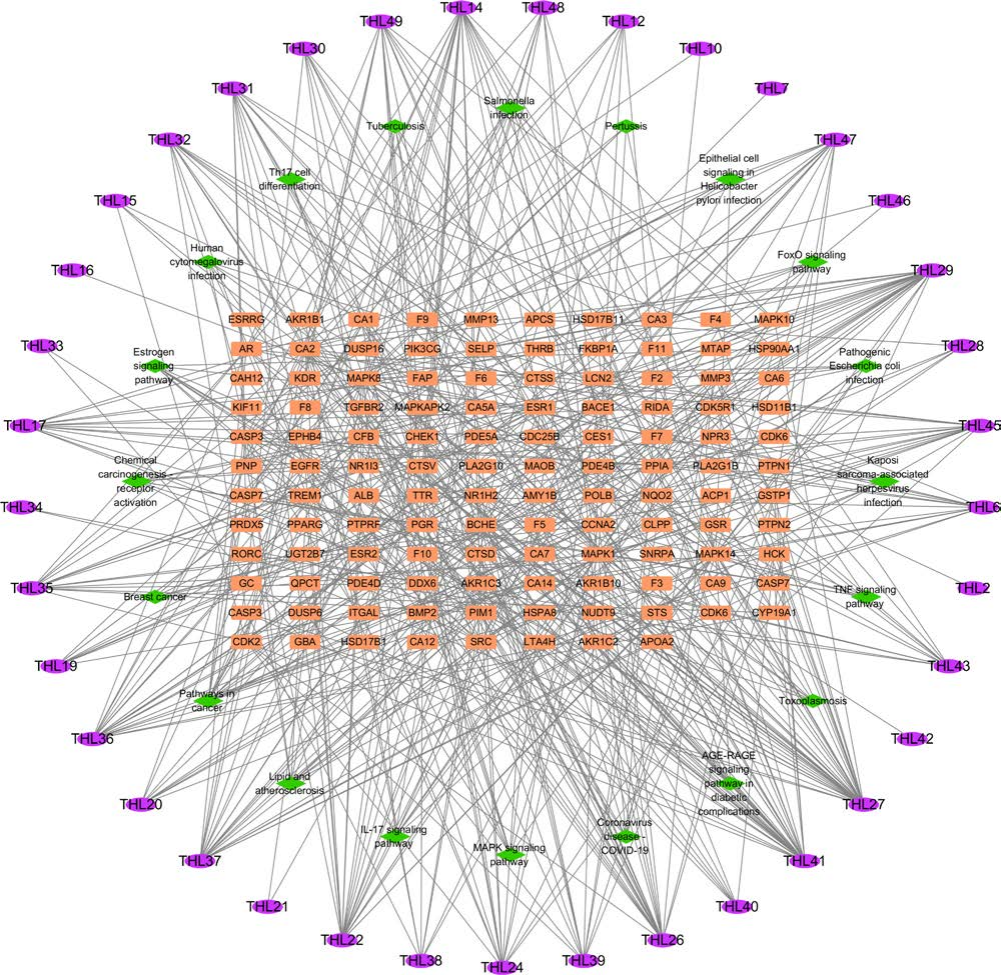
THL intervention pulmonary nodule component-target-pathway diagram. THL = *Polygonum capitatum* Buch.-Ham. ex D. Don.

CytoScape3.7.2 was used to construct the network diagram of “THL component-target-pathway,” and the topological information data of THL intervention PN disease target network were analyzed. Furthermore, the core chemical components and action targets were analyzed. Figure 5 reveals a network of 175 nodes and 577 edges. Orange nodes are potential targets of THL, green nodes are potential signal pathways in THL, purple nodes are active components of THL, and connecting lines symbolize the interaction between the different nodes.

Through the analysis of the network using Cytoscape, it was found that each active component of THL is linked to multiple targets, each of which links multiple components, suggesting that the multiple components within THL may be intervening PN through multiple targets. Among them, THL27 (quercetin) has a degree value of 39, an intermediate degree of 0.150, and a compactness of 0.418. It was predicted that THL27 was the main component of Polygonum capsuleum in the intervention of pulmonary nodules. Secondly, THL41 (hexadecane acid) has a connection degree of 39, intermediate degree of 0.265, and a tightness of 0.431. THL29 (leucine) has a connection degree of 25, a medium degree of 0.058 and a compact degree of 0.392 (Table 2). The network node degree of CA2, MAPK1, MAPK14, MAPK8, and BCHE target genes ranked high, with connectivity of 25, 25, 24, 21, and 16, respectively (Table 3). As shown in Table 4, different pathways are connected with each other through common targets, indicating that THL may play a synergistic role in intervening with PN through multiple pathways.

**Table 2:** Node characteristics of the main active ingredient network of THL.

Name	Degree	BetweennessCentrality	ClosenessCentrality
THL27	39	0.15006411	0.41849148
THL41	33	0.26459431	0.43107769
THL29	25	0.05824157	0.39179954
THL14	23	0.03820648	0.38478747
THL26	23	0.0808892	0.36673774
THL22	21	0.04780672	0.38478747
THL36	20	0.03814966	0.35908142
THL37	20	0.06053161	0.37636761
THL24	15	0.02915407	0.34331337
THL35	15	0.01558315	0.35463918

THL = *Polygonum capitatum* Buch.-Ham. ex D. Don.

**Table 3:** THL main active ingredient target network node characteristic parameters.

Name	Degree	Betweenness	ClosenessCentrality
CA2	25	0.16449539	0.44559585
MAPK1	25	0.07906922	0.40186916
MAPK14	24	0.08184698	0.40566038
MAPK8	21	0.03950728	0.3628692
BCHE	16	0.0448772	0.39814815
EGFR	16	0.03051126	0.34817814
ESR1	16	0.02279829	0.3553719
GSTP1	15	0.03838911	0.39631336
AR	15	0.03117308	0.39269406
PIM1	14	0.03400752	0.38565022

THL = *Polygonum capitatum* Buch.-Ham. ex D. Don.

**Table 4:** Enrichment results of THL intervention in pulmonary nodular target pathway.

GO	Description	Count	Gene ID
hsa05200	Pathways in cancer	17	AR BMP2 CASP3 CASP7 CCNA2 CDK2 CDK6 EGFR ESR1 ESR2 F2 GSTP1 HSP90AA1 PPARG MAPK1 MAPK8 TGFBR2
hsa05417	Lipid and atherosclerosis	11	CASP3 CASP7 MAPK14 HSPA8 HSP90AA1 MMP3 PPARG MAPK1 MAPK8 SELP SRC
hsa04915	Estrogen signaling pathway	9	CTSD EGFR ESR1 ESR2 HSPA8 HSP90AA1 PGR MAPK1 SRC
hsa04657	IL-17 signaling pathway	8	CASP3 MAPK14 HSP90AA1 LCN2 MMP3 MMP13 MAPK1 MAPK8
hsa04610	Complement and coagulation cascades	7	CFB F2 F3 F5 F7 F8 F9
hsa05207	Chemical carcinogenesis - receptor activation	9	AR EGFR ESR1 ESR2 HSP90AA1 PGR MAPK1 SRC NR1I3
hsa05215	Prostate cancer	7	AR CDK2 EGFR GSTP1 HSP90AA1 MMP3 MAPK1
hsa01522	Endocrine resistance	7	MAPK14 EGFR ESR1 ESR2 MAPK1 MAPK8 SRC
hsa05161	Hepatitis B	8	CASP3 CCNA2 CDK2 MAPK14 MAPK1 MAPK8 SRC TGFBR2
hsa04914	Progesterone-mediated oocyte maturation	7	CCNA2 CDK2 MAPK14 HSP90AA1 PGR MAPK1 MAPK8

THL = *Polygonum capitatum* Buch.-Ham. ex D. Don.

### 3.6. THL molecular docking screening

**Figure 6.**
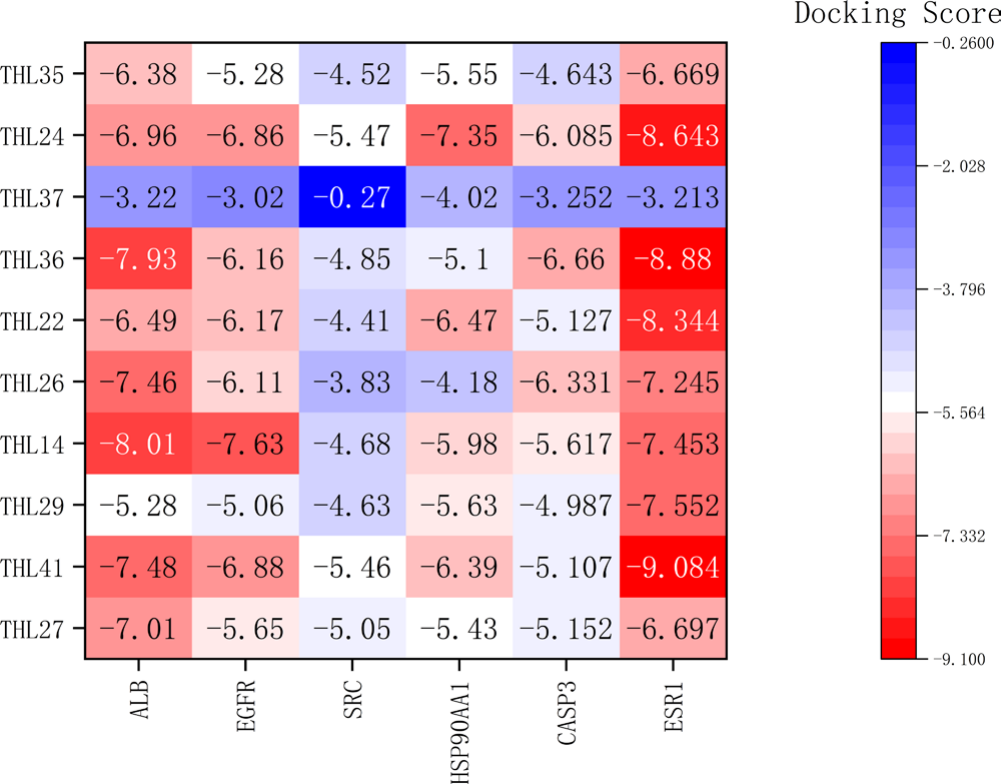
Molecular docking results.

**Figure 7.**
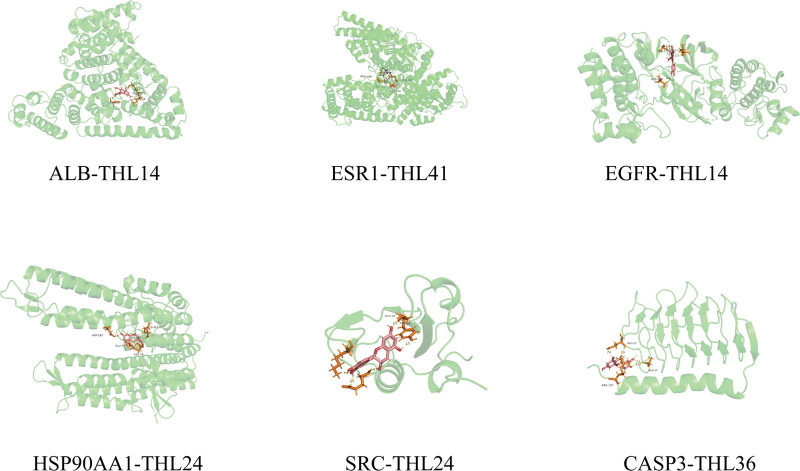
Molecular docking mode of some core compounds of THL(3D). THL = *Polygonum capitatum* Buch.-Ham. ex D. Don.

**Figure 8.**
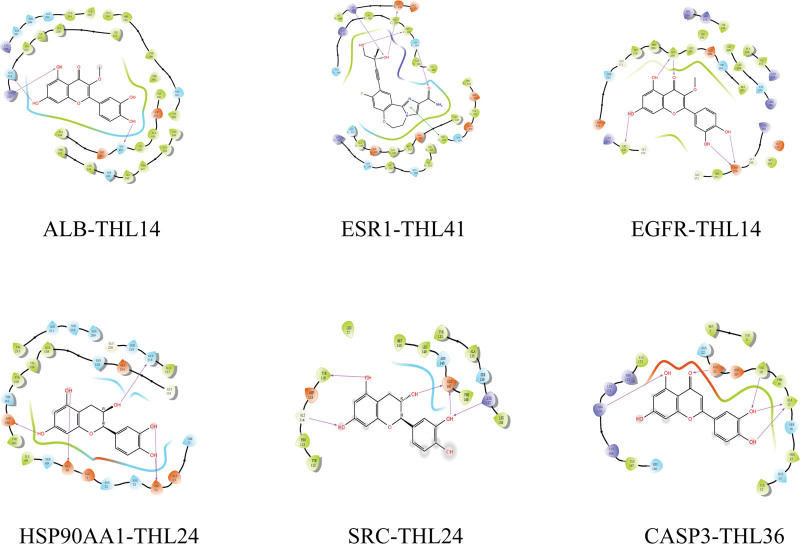
Molecular docking mode of some core compounds of THL(2D). THL = *Polygonum capitatum* Buch.-Ham. ex D. Don.

Perform molecular docking between the top 6 targets with moderate values in PPI network results and the top 10 active compounds. Obtain protein PID from the RCSB database and import it into Maestro software for molecular docking with core components. The results are represented by Docking Score. Select the core docking results based on Docking Score and use Pymol software for visualization processing. The docking results are shown in Figure 6, and the core docking modes are shown in Figures 7 and 8.

### 3.7. Ethical approval

The current analysis does not require ethical approval, because our analysis only collects uploaded data information from the public database search.

## 4. Discuss

### 4.1. Analysis of potential active components

The main active ingredients of THL in treating PN are Quercetin, Salidroside. Quercetin, a natural flavonoid, has been discovered to have anti-inflammatory, antibacterial, antioxidant, anti-cancer, and other biological properties.^[4]^ The occurrence of PN is mostly attributed to chronic lung inflammation, and then it may develop into malignant lung lesions. As confirmed by studies, TNF plays a significant role in PN inflammatory lesions and inhibiting diffusion.^[5]^ Inhibition of oxidative stress and inflammatory reaction by quercetin impede the production of inflammatory mediators, thereby influencing the TNF signaling pathway. Quercetin can effectively hinder tumor cell multiplication, induce programmed cell death, suppress tumor cell migration and invasion, which may be related to PI3K/Akt/mTOR signaling pathway, Wnt/β-catenin pathway and MAPK/ERK1/2 pathway.^[6]^ Salidroside, as one of the main effective components extracted from Rhodiola rhizome, has pharmacological effects such as antioxidant stress, anticancer and reducing inflammatory reaction. The inhibition of NF-κB and NACHT; LRR and PYD domains-containing protein 3 (NLRP3) signaling pathways by Salidroside, as well as the decrease of pro-inflammatory factors such as IL-1β, IL-6, and TNF-α, can help to reduce the pulmonary inflammatory reaction of acute PN in rats.^[7]^ In vitro, salidroside can inhibit activation of NLRP3 inflammatory corpuscles and subsequent caspase-1 lysis and interleukin (IL)-1β secretion.^[8]^ The capacity of salidroside to suppress oxidative stress response in various cells, inhibiting tumor migration induced by transforming growth factor TGF-β as well as the development of cancer cells and causing them to stagnate in G0/G1 phase. Moreover, by decreasing the phosphorylation level of p38 and the amount of reactive oxygen species in cells, the expansion of A549 lung cancer cells is inhibited.^[9]^ Salidroside, in addition, has the capacity to augment Bax expression and diminish Bcl-2 expression in tumor tissues, thus hindering the proliferation of tumor cells, inducing their apoptosis, and give play to an anti-tumor effect. As a pentacyclic triterpenoid, Oleanolic acid was first isolated from the leaves of olea europeae L by British FB Power in 1908. Pharmacological effects of it include anti-inflammation, anti-oxidation, and anti-tumor. The activation of Toll like TLR4 and PI3K/AKT signaling pathways mediates inflammation and oxidative stress, thus playing a crucial role in the genesis and development of PN. Oleanolic acid can reduce the expression of TLR4 protein, regulate the PI3K/AKT signaling pathway, and thus impede the release of inflammatory factors and oxidative stress response.^[10]^ The induction of inflammatory cytokines is linked to NLRP3 inflammatory corpuscles, and oleanolic acid has been found to impede their activation and reduce the expression of IL-1β and IL-18, thus alleviating the inflammatory response of mice lung.^[11]^ At the same time, miR-122 refers to an important tumor suppressor.The expression of miR-122 is significantly increased by oleanolic sour, and hinder the proliferation of cancer cells.^[12,13]^

### 4.2. Target analysis

As shown by the results of network topology analysis, there were 49 active components of THL, corresponding to 114 targets, among which 67 common targets related to PN. By constructing PPI network, it could be found that ALB, EGFR, ESR1, HSP90AA1, etc. have large degrees values, which may be the most critical therapeutic targets for THL. In addition, ALB is a multifunctional protein molecule whose main functions are anti-oxidation regulation of inflammation and immune response. The incident and advancement of neoplasms are often linked to inflammation and the body state of the patient. ALB can predict the occurrence and development of PN, reflecting the nutritional status and possible inflammatory reactions in the patient’s body. Abnormal expression of EGFR has a major influence on the growth and development of tumors, and anti-EGFR antibodies have been observed to significantly decrease the number of PN mice.^[14]^ Tyrosine kinase inhibitors can impede the self phosphorylation of EGFR, and abnormal tumor multiplication prevented.^[15]^ HSP90AA1 is a gene that is highly expressed in lung cancer patients and is known to promote squamous cell lung cancer. In vitro, siRNA was used to silence HSP1AA1 gene and induce apoptosis of A549 cells. As shown by studies,^[16]^ reducing the expression of HSP90AA1 can reduce inflammatory factors, reactive oxygen species production, apoptosis rate and autophagy-related proteins. ESR1 is a gene encoding estrogen receptor alpha, whose mutation is closely related to cancer,^[17]^ inflammation^[18]^ and other pathological processes. Studies have demonstrated that XCT-790,^[19]^ an ERR α reverse agonist, can decrease the expression of IL-8 in cancer cells and impede their proliferation and migration.

### 4.3. Bioenrichment analysis

An examination of GO function enrichment uncovers that THL intervention in PN is a biological process involving hormone response, cellular response to organic cyclic compound, intracellular receptor signaling, hormone-induced signaling, and cellular response to lipid, etc. As shown by KEGG signal pathway analysis, THL intervention in PN involves various pathways in cancer, lipid and atherosclerosis, IL-17 signaling path, etc. The occurrence of PN is closely linked to lipid and atherosclerosis. A serious disruption of amino acid, lipid and cholesterol metabolism in the plasma of both benign and malignant PN patients was revealed through a plasma metabonomics and lipomics analysis.^[20]^ Lipid metabolism indexes are of great clinical significance in the diagnosis of both benign and malignant PN, and fatty acids may serve as serum markers for malignant PN.^[21]^ Regulating lipid metabolism, inflammation,^[22]^ oxidative stress,^[23]^ and tumor cell growth can be inhibited, thus having a part to play in treating PN. IL-17 signaling pathway is a key pathway involved in PN formation and aggregation of inflammatory cells in lung. Cytokines of the IL-17 family can activate a range of immune signaling molecules to induce the release of inflammatory factors, such as IL-17, IL-1β, IL-6, and TNF-α. At the same time, the IL-17 family is not only a pro-inflammatory cytokine, but also pro-tumor cytokine. Confirmed by numerous studies, IL-17, a pro-tumor cytokine, can be a catalyst for cancer’s emergence by stimulating cell proliferation, creating blood vessels, and intensifying the aggregation and activation of inflammatory cells.

In conclusion, THL can not only treat PN through cancer, lipid and atherosclerosis, and IL-17 signaling pathway, but also interfere with the development of PN to cancer. Therefore, THL can not only treat PN, but also reduce the risk of cancer in PN patients, playing a positive preventive role.

## 5. Summary

The etiology of PN is unclear, in which tumor is one of the properties of PN. The current diagnosis and treatment focus on lessening the transition treatment of benign nodules, as well as enhancing the quality of life for patients. As a traditional officinal botany, THL has been widely used for its antibacterial, anti-inflammatory and antioxidant activities.^[24]^ However, there are few studies on its treatment of PN. Utilizing network pharmacology and molecular docking technologies, this study initially elucidated THL’s mechanism of multi-component and multi-target combined regulation of pulmonary nodules, utilizing target prediction, protein interaction network, GO enrichment analysis, and KEGG pathway enrichment analysis. THL may act on ALB, EGFR, HSP90AA1, ESR1, and other biological targets through quercetin, salidroside, oleanolic acid and other active components, participate in many signal pathways such as Pathways in cancer, Lipid and atherosclerosis, IL-17 signaling pathway to intervene with PN, and play anti-tumor, anti-cancer cell proliferation, anti-inflammation, anti-oxidation, etc. This study is based on scientific theories to speculate on the mechanism of THL intervention in PN, providing a theoretical basis for its secondary development. This study, though based on the calculation results of various biological information databases and big data, was limited by the limitations of network pharmacology research without did not include any possible changes in preparations and drug ingredients during administration process into the analysis. Consequently, it is still necessary to conduct animal or cell experimental studies to further investigate the mechanism of action of THL intervention in PN. To provide more effective treatment guidelines for PN prevention and treatment with traditional Chinese medicine.

## Author contributions

**Conceptualization:** Xiang Pu, Chen Zhang.

**Data curation:** Lailai Li.

**Formal analysis:** Xiang Pu.

**Funding acquisition:** Xiang Pu.

**Investigation:** Xiang Pu.

**Methodology:** Lailai Li.

**Resources:** Zhiliang Fan, Xiangyan Kong.

**Software:** Zhiliang Fan.

**Validation:** Yilian Tang.

**Visualization:** Yilian Tang, Chen Zhang.

**Writing – original draft:** Yilian Tang.

**Writing – review & editing:** Yilian Tang.
